# Phosphorylation promotes binding affinity of Rap-Raf complex by allosteric modulation of switch loop dynamics

**DOI:** 10.1038/s41598-018-31234-7

**Published:** 2018-08-28

**Authors:** Devanand T, Prasanna Venkatraman, Satyavani Vemparala

**Affiliations:** 1 The Institute of Mathematical Sciences, C.I.T. Campus, Taramani, Chennai, 600113 India; 2 ACTREC, TMC, Sector 20, Kharghar Navi Mumbai, 410210 India; 3 Homi Bhabha National Institute, Training School Complex, Anushakti Nagar, Mumbai, 400094 India

## Abstract

The effects of phosphorylation of a serine residue on the structural and dynamic properties of Ras-like protein, Rap, and its interactions with effector protein Ras binding domain (RBD) of Raf kinase, in the presence of GTP, are investigated via molecular dynamics simulations. The simulations show that phosphorylation significantly effects the dynamics of functional loops of Rap which participate in the stability of the complex with effector proteins. The effects of phosphorylation on Rap are significant and detailed conformational analysis suggest that the Rap protein, when phosphorylated and with GTP ligand, samples different conformational space as compared to non-phosphorylated protein. In addition, phosphorylation of SER11 opens up a new cavity in the Rap protein which can be further explored for possible drug interactions. Residue network analysis shows that the phosphorylation of Rap results in a community spanning both Rap and RBD and strongly suggests transmission of allosteric effects of local alterations in Rap to distal regions of RBD, potentially affecting the downstream signalling. Binding free energy calculations suggest that phosphorylation of SER11 residue increases the binding between Rap and Raf corroborating the network analysis results. The increased binding of the Rap-Raf complex can have cascading effects along the signalling pathways where availability of Raf can influence the oncogenic effects of Ras proteins. These simulations underscore the importance of post translational modifications like phosphorylation on the functional dynamics in proteins and can be an alternative to drug-targeting, especially in notoriously undruggable oncoproteins belonging to Ras-like GTPase family.

## Introduction

Rap belongs to the family of small Ras-like GTPases, which have many roles in cellular activities like cell proliferation, apoptosis and differentiation etc.^1–5^. These GTPases act like molecular switches, active when GTP is bound and inactive when GDP is bound. These conformations are interconvertible by the action of the guanine nucleotide exchange factors (GEFs) and The GTPase activating proteins (GAPs). GEFs exchange GDP for GTP and GAPs catalyse the hydrolysis of GTP converting the active form into the inactive protein^6^. The Ras GTPases participate in many signalling pathways, including the MAPK/ERK, PI3K^7–9^. Many factors including their cellular location, bound ligand molecule and phosphorylation can affect how these molecules interact with downstream signalling proteins^10,11^, which is crucial in transmitting signal from Ras to the mitogen-activated protein kinase. Because of their role in key signalling events which are often deregulated in cancer and due to their prominent role as oncogenes, Ras family members (specifically H-, K- and N-Ras proteins) have garnered considerable attention over the years^9,12–14^. Considering all cancers where at least 20 tumours were counted and weighted equally, pan ras mutations were found at an incident rate of 16%^14^ and activating Ras mutations are associated with approximately 30% of all human cancers^8^. Many of these mutations render the tumour aggressive and are responsible for the death of patients. Yet there are no targeted therapies for these class of proteins as they are considered notoriously undruggable lacking specific binding pockets^13^.

The Rap proteins such as the Rap1A and Rap1B rose to prominence because of their high degree of identity to the Ras proteins^1,4,15–19^. Rap1A was identified as a suppressor of Ras activity in screening assays, a function attributed to its ability to competitively bind (in the presence of GTP) to downstream Raf without activating it and hence disrupting the signal transmission along the MAPK pathway^20^. Binding of Rap1 to RafB on the other hand results in activation as seen with the Ras family of proteins^21^. Many mutations, domain swapping experiments have indicated regions other than the RBD domain of Raf are responsible for these differences^22,23^. All known Ras effectors share a common Ras-binding Domain (RBD). Besides competing with Ras, Rap proteins are involved in many other crucial cellular functions such as cell adhesion, cell-cell junction formation and regulation of the actin cytoskeleton^24–27^.

Although not as well studied as the role of GEF proteins and GAP catalyzed changes in the nucleotide bound conformations, phosphorylation is known to regulate the functions of Ras and Rap proteins^28–32^. Phosphorylation is the most common reversible post translational modification(PTM) of proteins with a role in regulation of essentially all cellular functions^33,34^. The mechanism of how phosphorylation acts as a molecular switch that allows cells to respond instantaneously to various stimuli without the need for new protein synthesis, how phosphorylation at a remote site often influences the activity at a completely different site continue to be active areas of research. Most often phosphorylation is observed in disordered, well accessible or highly flexible regions and loops in proteins^35,36^. However, multiple investigators, including the author of this paper, while analyzing large scale data sets have documented that many of these phosphosites are not readily exposed to the solvent^36–39^. The presence of significant number of possible phosphosites in the disallowed region of phosphoconformation suggests that factors including dynamic conformational changes of proteins, binding to other proteins or regulatory factors can potentially expose these buried phosphosites to the solvent and to a kinase. Therefore understanding the role of protein dynamics in exposing such sites to solvents, interactions involved in transmitting the effects of phosphorylation to other functional sites is essential. Inherent loop dynamics of proteins are known to play a critical role in functioning of the protein^40–43^. Local mutations and PTMs impact the local loop dynamics and global structure and function of the protein. However it is not trivial to obtain phosphoproteins in amounts large enough for experimental investigations even when the kinase involved is known. MD simulations are invaluable tools successfully employed on multiple occasions to understand the effect of phosphorylation on the structure, dynamics, allosteric effect, conformational stabilization and map the electrostatic interactions in the proteins and thereby deduce the effect on functions^44–48^.

**Figure 1:**
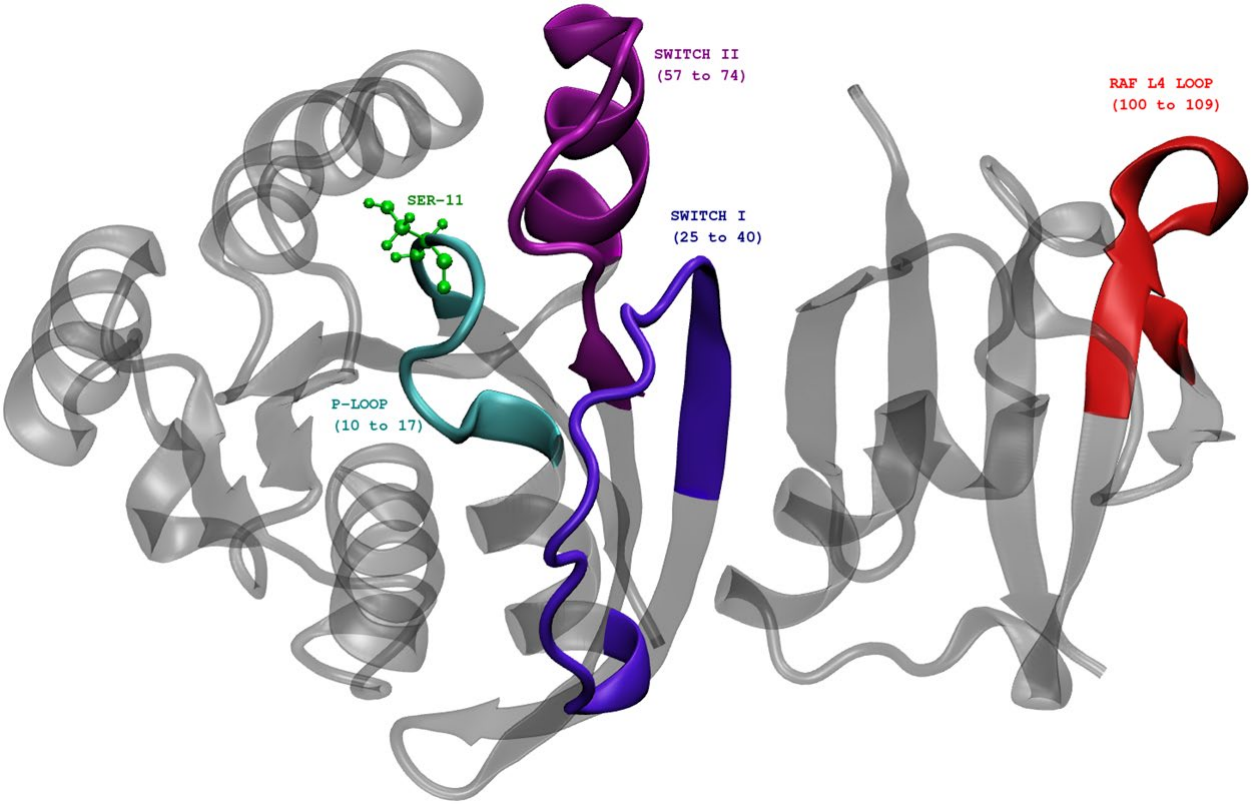
Rap-Raf protein complex (crystal structure, PDB ID 1C1Y) showing the location of important functional loops like P-loop, Switch I, Switch II and RBD loop regions. The phosphosite SER11 is shown in green.

In this paper, we study the effects of phosphorylation of a single residue, SER11, identified as a possible phosphosite in Rap1A (Supplemental Table 1 of ^37^ and Table S1 of ^49^) (see Fig. 1), on the conformational dynamics of the Rap1A and its interactions with the effector protein kinase c-Raf1. It is to be noted that this particular residue, serine, occurs only in Rap GTPases and is replaced by alanine in Ras GTPases^50^. Phosphorylation at the same site has been observed in tumor samples of lung cancer patients as well (see Supplemental table of ^51^) and SER11 phosphosite in RAP1 carries motifs for many kinases, some with high and others with moderate scores: for example putative sites CK1, Aurora and ATM kinases, are predicted by KinasePhos2 a webserver for phosphosite predictions^52^. In addition to SER11 residue, SER39, SER179 and SER180 are other possible phosphosites in Rap1 that are either predicted or experimentally determined. Proximity of SER11 to the nucleotide ligand, which alters the activity of GTPases such as Rap1, renders the investigation of effects of phosphorylation particularly interesting. We also explore the effects of such phosphorylation on the dynamics of functional loops such as Switch I and Switch II loops to characterize the allosteric pathways within Rap-Raf complex and subsequently gain some insight into possible mechanisms through which Rap may affect the downstream MAPK signalling pathway.

## Results

### Effects of phosphorylation on structure and dynamics of Rap

**Figure 2:**
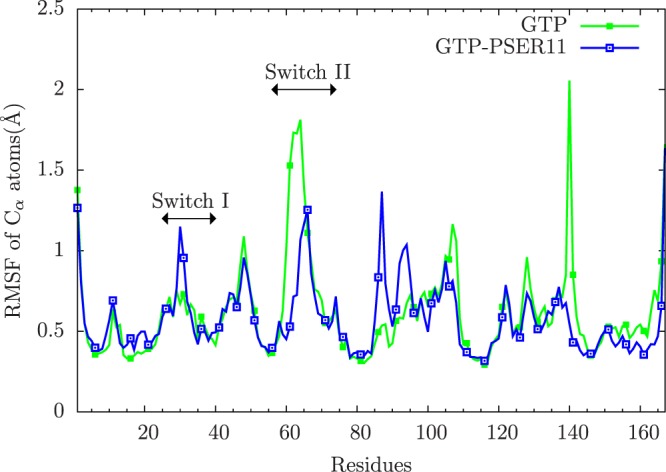
The RMSF of residues of Rap C_*α*_ atoms averaged over 350 to 400 ns of simulation for both unphosphorylated (green) and phosphorylated (blue). The relevant Switch I and Switch II loops are indicated in the figure.

The stability of the structures monitored via the root mean squared deviation of the GTP-bound Rap protein with and without the SER11 phosphorylated is shown in Fig. S1. The mobility of various parts of the protein and the effect of phosphorylation on the mobility of the Rap protein is measured through the root mean squared fluctuations (RMSF) of each residue, averaged over last 50 ns, 350–400 ns (Fig. 2). In the GTP bound form, the effects of SER11 phosphorylation are centered around the Switch I and Switch II loop regions. Proteins belonging to Ras superfamily are known to inhabit multiple conformational states and the two important loops that determine such conformational flexibility are Switch I (residues 25–40) and Switch II (residues 57–74) loops. The mobility of the loop region containing residues 80–85, which is spatially proximal to the phosphosite, is increased in the case of phosphorylated SER11. This is due to the acquired favourable interactions between the phosphorylated SER11 and the Switch II loop (discussed later) and consequently disrupt the interaction between the phosphosite and the loop containing residues 80–85. To understand the effects of complex formation on the structure of Rap, we also simulated the Rap protein alone (not in complex with Raf), with GTP ligand. The RMSF plots for all the four cases of Rap protein with or without complex formation with RBD of Raf protein and with and without phosphorylation of SER11 is shown in Fig. S2. The results suggest that the loop with residues 138–141 is mobile in all cases except the case of Rap-Raf complex with SER11 phosphorylated. This is due to favourable electrostatic interactions between ASN140 and ASP108. Results (Fig. S2) suggest that the mobility of both switch loops is very high when Rap is not in complex with RBD of Raf but undergoes reduction of mobility when SER11 is phosphorylated. The Solvent accessible surface area (SASA) of SER11 residue calculated for the molecular dynamics trajectory of Raf-Rap complex with GTP (measured using VMD keeping the probe radius to 1.4 Å) indicates that the dynamical nature of the P-loop allows the buried phosphosite SER11 to be exposed to water for a significant time on the present simulation timescale, suggesting a strong possibility of a kinase phosphorylating the SER11 residue and lends validity to our simulation studies involving phosphorylated Rap protein (Fig. S3). In the following sections we describe how phosphorylation at SER11 influences the dynamics that affects a) Switich I loop interaction with nucleotide and RBD domain and b) Switch II loop interaction with the nucleotide.

**Figure 3:**
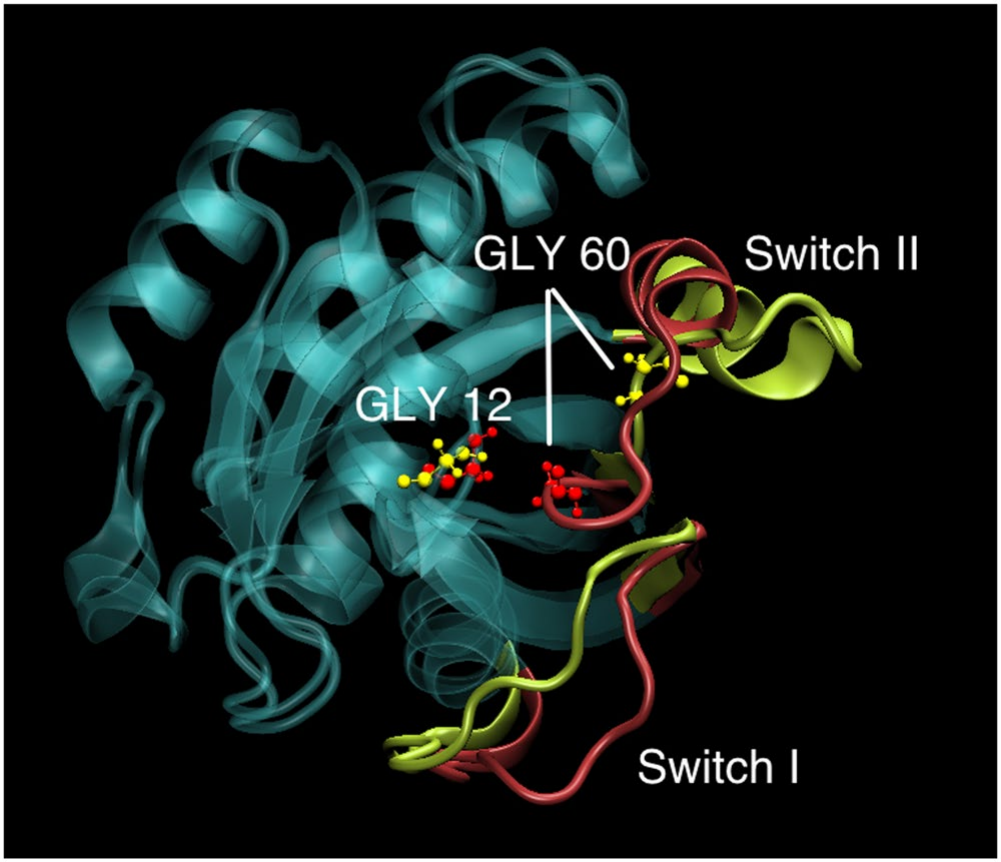
Overlapped snapshots of GTP bound Rap protein showing the conformation of functional Switch I and Switch II loops with (red) and without (yellow) phosphorylation towards the end of the simulation. The positions of residues GLY 12 and GLY60 with (red) and without(yellow) phosphorylation is also marked.

**Figure 4:**
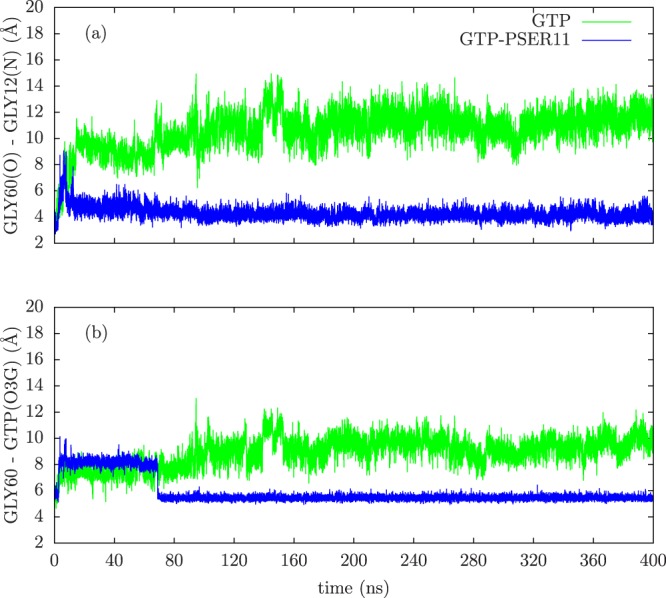
The time evolution of (**a**) distance between GLY60(O) and GLY12(N) atoms, (**b**) distance between GLY60(center of mass) and GTP(O3G) atoms for both unphosphorylated (green) and phosphorylated (blue) cases.

Switch II loop interacts primarily with the GDP/GTP exchange factors (known as GEFs), which accelerate the release of the previously bound GDP to the proteins and to be replaced by GTP. The conformation of Switch II loop undergoes profound changes when GDP is exchanged with GTP ligand^6,53^. The GEF proteins are known to make extensive contacts with residues in the Switch II loop inducing local conformational changes near the nucleotide binding site, which results in the release of the bound nucleotide. Thus the mobility of the Switch II loop plays an important role in binding the GEF proteins leading to the GTP-bound conformations of G-proteins and consequently affect their ability to bind to downstream effectors. From the Fig. 2, it can be seen that the Switch II loop is most mobile in the GDP bound form of Rap, whose mobility reduces in the GTP bound form. This can be understood as the conformational stability that the loop acquires upon its interaction with the GEF proteins. Phosphorylation of the SER11 residue further reduces the mobility of this important functional loop in the GTP bound form of the protein. To understand this difference in the dynamics of the Switch II loop, its interactions with residues in the nearby P-loop (to which SER11 residue belongs) were investigated. In the Rap protein, as with the Ras proteins, the nucleotide pocket is flanked by primarily three loops: Switch I, Switch II and P-loop. As can be seen in Fig. 3, the conformation of Switch II loop is drastically altered when SER11 is phosphorylated in Rap-GTP protein. There are two strong electrostatic interactions which underlie such a significant conformational change. The Switch II loop contains several polar and charge residues including ARG68, which is positively charged. On phosphorylation of the SER11, the pocket region close to this residue acquires more negative charge compared to the unphosphorylated Rap. The simulations strongly suggest there is a phosphorylation induced change in conformation of Arg 68, which forms a stable salt bridge with the phosphate group of the SER11. This strong interaction results in pulling of the Switch II region into the nucleotide binding pocket which results in formation of another stable electrostatic interaction between the main chain carbonyl oxygen and the amide nitrogen atoms belonging to GLY 60 (Switch II) and GLY 12(P-loop) respectively. The distance between the two residues in the Rap-GTP protein with and without phosphorylation throughout the simulation timescale are shown in Fig. 4(a) and the difference is almost of the order of 7 Å. The GLY60 residue also forms stable favourable interactions with the oxygen atoms of the GTP ligand when SER11 is phosphorylated (Fig. 4b). The resulting favourable electrostatic interaction locks the Switch II loop into a conformation that reduces drastically its mobility.

**Figure 5:**
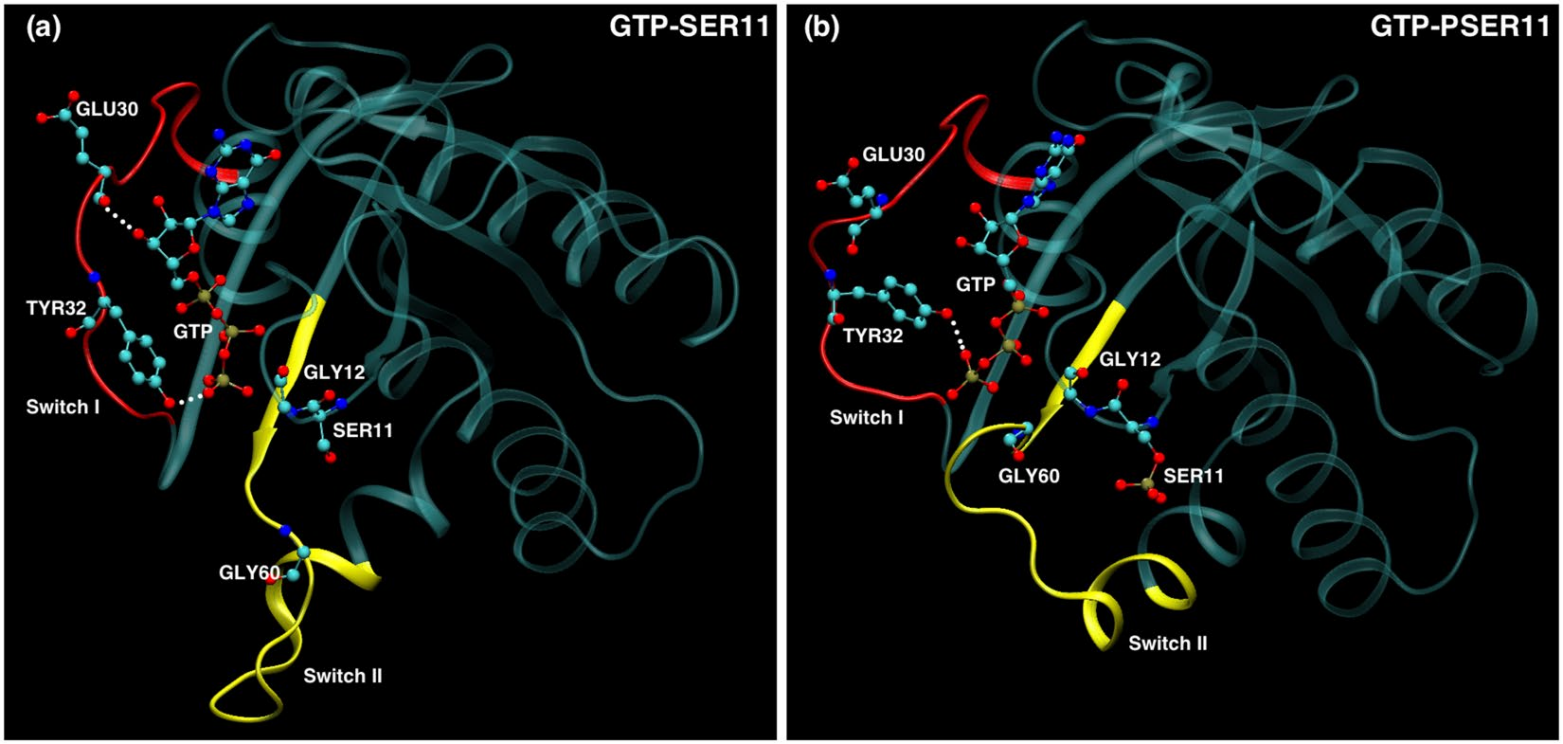
Snapshots of nucleotide pocket of Rap-GTP with and without phosphorylation of SER11. The functional loops Switch I and Switch II loops are shown in red and yellow colour respectively. The relevant residues in the two loops are also shown and the hydrogen bonds between GTP and residues in Switch I loop, when relevant, are shown in white dashed lines.

Switch I loop of Rap interacts directly with the effector protein Raf kinase, and the strength of the interaction strongly depends on the bound nucleotide. Compared to the inactive state of GDP bound Ras, RBD binds to active GTP bound Ras, almost 1000 times more strongly^54^. This mode of interaction is conserved in Ras superfamily of proteins^10,55,56^. Hence, the dynamic mobility of the Switch I loop is of crucial importance in the interaction between proteins in Ras superfamily and their effectors. The RMSF plot in Fig. 2 shows that the Switch I loop has slightly increased mobility when SER11 is phosphorylated, compared to the unphosphorylated Rap-GTP protein (in the GTP bound forms). The increased mobility is largely due to the movement of Glu30 of Switch I loop region (Fig. 5(a)). In the unphosphorylated Rap-GTP case, the GTP ligand forms several favourable interactions with residues lining the nucleotide pocket. This includes a strong and persistent hydrogen bond between the the oxygen molecules attached to the *γ*-phosphate atom and the hydroxyl group of TYR32, which has been observed in many crystal structures of Ras super family including Rap^57,58^. The other favourable electrostatic interaction of GTP ligand with Switch I loop is between GLU 30 and hydroxyl groups attached to ribose moieties of GTP ligand (see Fig. 5). These interactions reduce the mobility of the Switch I loop and participate in the stability of the complex formation with RBD loop of Raf kinase. With the inward movement of the Switch II loop into the nucleotide pocket region, as mentioned above, the position of GTP ligand changes and the interaction between GLU30 and GTP ligand is broken (see Fig. 5(b)), resulting in increased mobility of the residue GLU30 (see Fig. 2). It is to be noted that the position of TYR32 in the crystal structure used in the present simulations (1C1Y) is in the conformation in which the residue is located within the active site^59,60^. This conformation of TYR32 is expected to play a crucial role for catalysis of Rap proteins^61^, independent of GAP proteins, and that this conformation of TYR32 is preserved even when nucleotide pocket is significantly perturbed when SER11 is phosphorylated. This alteration of dynamics of Switch I loop by phosphorylation of SER11, which is located spatially and sequentially away from Switch I, clearly shows that allosteric mechanism is involved in communication.

**Figure 6:**
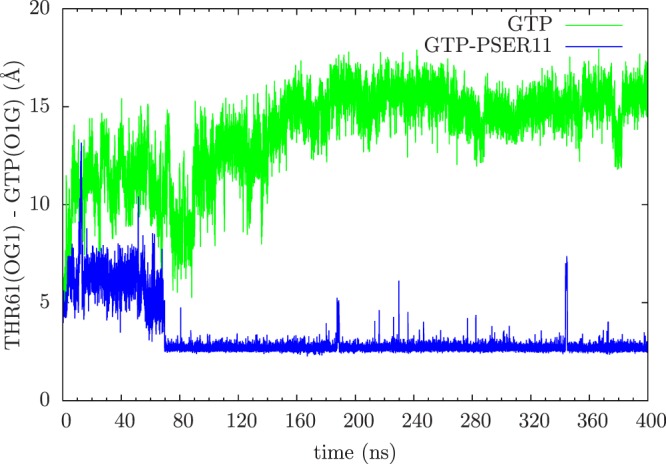
The time evolution of distance between the catalytic residue THR61 and GTP ligand without (green) and with (blue) phosphorylation.

The most significant effect of phosphorylation of SER11 is in the position of THR61 residue on the Switch II loop, with respect the bound ligand GTP as shown in Fig. 6. In Ras proteins, the residue 61 (which is GLN) plays a very crucial role, along with the GAP proteins, in the GTP hydrolysis. The residue 61 is one of the most mutated site found in human tumours which impairs or abolishes the hydrolysis of GTP can lend the Ras protein to be in perpetually ON state^14^. In Rap proteins, this important residue is replaced by a threonine and experimental studies have shown that THR61, unlike GLN61 in Ras, plays a predominant role in binding of GAP proteins and does not participate in GTP hydrolysis^59,62^. The solved structure of Rap1 in complex with Rap1GAP has shown that the conformation of THR61 is away from the active site^59^. In the Rap-RBD structure used in the present simulation also, the Switch II is in a disordered state and THR61 is pointed away from the nucleotide. The simulations with the unphosphorylated Rap-RBD complex shows that the Switch II loop remains mobile and the THR61 moves away from the nucleotide during the course of the simulation. However, the phosphorylation of SER11 residue brings the THR61 into the nucleotide active site by forming a stable bond with GTP (as seen in Fig. 6). This conformation and location of THR61 inside the active site can have profound effect on the ability of the GAP proteins to hydrolyse the GTP ligand and can potentially affect Rap’s interaction with Raf.

### Conformational sampling of Rap: Effects of phosphorylation

**Figure 7:**
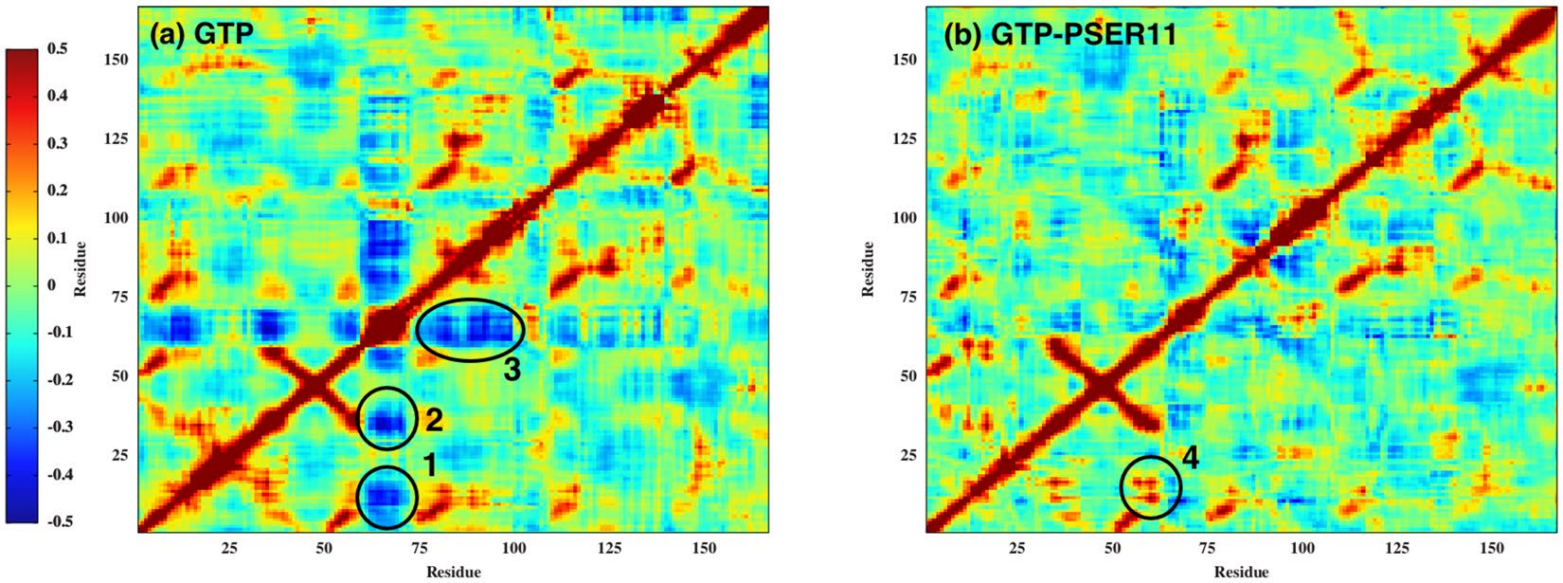
Cross-correlation plots of the Rap complex for 300 to 400 ns trajectory data of GTP- and GTP-PSER11 cases.

Having found that there is a reciprocal relationship in the dynamics of the switch loops that has a strong influence on nucleotide binding and Raf interaction the role of phophorylation on the conformation of different forms of the protein and effect on Rap activity is further explored in this section. Covariance analysis, using the cross correlation matrix as defined in the Methods section, is a very useful tool in getting insights into the relative correlated motions of different parts of the protein. The cross correlation matrix is computed by measuring the positional deviations of individual residues from an averaged structure and it is further averaged over equilibrium trajectory time scale. For all the simulations considered in this study, the cross correlation matrix is constructed over last 100 ns of simulation time in a run of 400 ns. We would like to emphasise that in our simulations, all rotations and translations were removed before performing the cross correlation analysis, as is the norm. The presence of hinges and possible large scale movement about the hinges can potentially complicate the positional cross correlation measurements, but no such global changes have been observed in all our simulations. It is very evident from the results (shown in Fig. 7) that the phosphorylation significantly alters the correlation between various functional loops. In the unphosphorylated Rap, the Switch II loop is anticorrelated with both Switch I and P-loop (shaded in blue 1and 2 respectively in Fig. 7), which disappears in the case of phosphorylated Rap protein. In addition, a strongly positively correlated motion (region 4 in Fig. 7) appears between Switch II and P-loop region. These results are consistent with the observations made in the previous section: the attractive interactions between the GLY60 in Switch II loop and GLY12 in P-loop triggers the observed positively correlated motion between the two loops. The conformational change in Switch II loop also removes strong anti-correlation between Switch II and helix 4 (residues 75 to 100) in Rap (region 3 in Fig. 7), further suggesting an overall increase in the correlated motion between different parts of the Rap protein when SER11 is phosphorylated.

**Figure 8:**
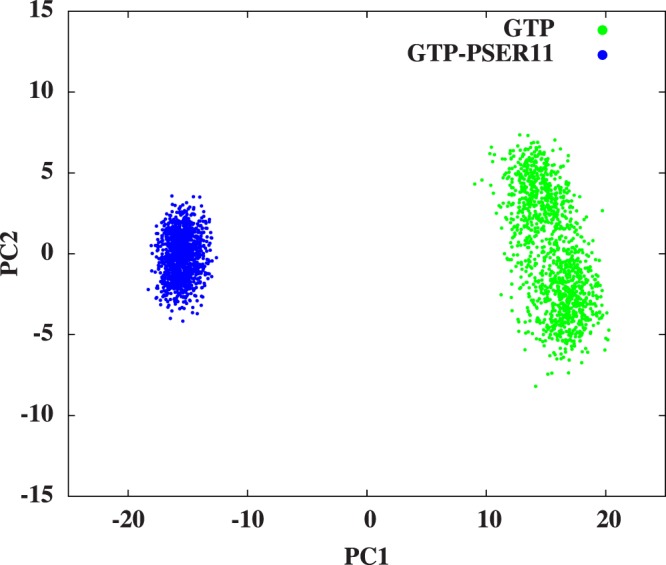
Conformer plots of Rap and Raf domains respectively(375 to 400 ns simulation data). The plot shows conformational space sampled by Rap protein in terms of PC1(80.13%) and PC2(2.36%).

To understand the change in configurational space explored by the Rap protein when SER11 is phosphorylated, PCA analysis is employed. As has been described earlier, the two major regions which experience considerable changes after phosphorylation are localized regions in Switch I and Switch II loops. It has been long proposed that the proteins involved in the complex formation undergo changes in conformational entropy to compensate for the loss of translational entropy, due to complex formation, and it would be interesting to see if the phosphorylation can affect such conformational sampling. The mobility of the Switch II loop is considerably reduced when SER11 is phosphorylated and the loop’s configuration also changes such that the residues on the loop are pulled significantly towards the nucleotide binding site. The Switch I experiences an increase in its mobility on phosphorylation, but the change is much less compared to the reduction of mobility of Switch II loop. From these results, it can be expected that the Rap molecule acquires an overall tighter configuration on phosphorylation which can be verified through monitoring the subspace defined by the two largest principal components (PC1 and PC2) of the projected MD trajectory. Towards this we have analyzed the trajectory data generated over the last 25 ns of MD simulations (375–400 ns) by fitting the coordinates of all the frames of C_*α*_ atoms. The results clearly show that the Rap protein occupies a different conformational space compared to the unphosphorylated form and that the over all conformational density is smaller, strongly indicating a tighter conformation (see Fig. 8). The first two principal component vectors PC1 and PC2 captured nearly 83% of the information content from the last 25 ns data of MD trajectories of Rap domain C_*α*_ atoms for the GTP liganded forms with and without SER11 phosphorylation.

**Figure 9:**
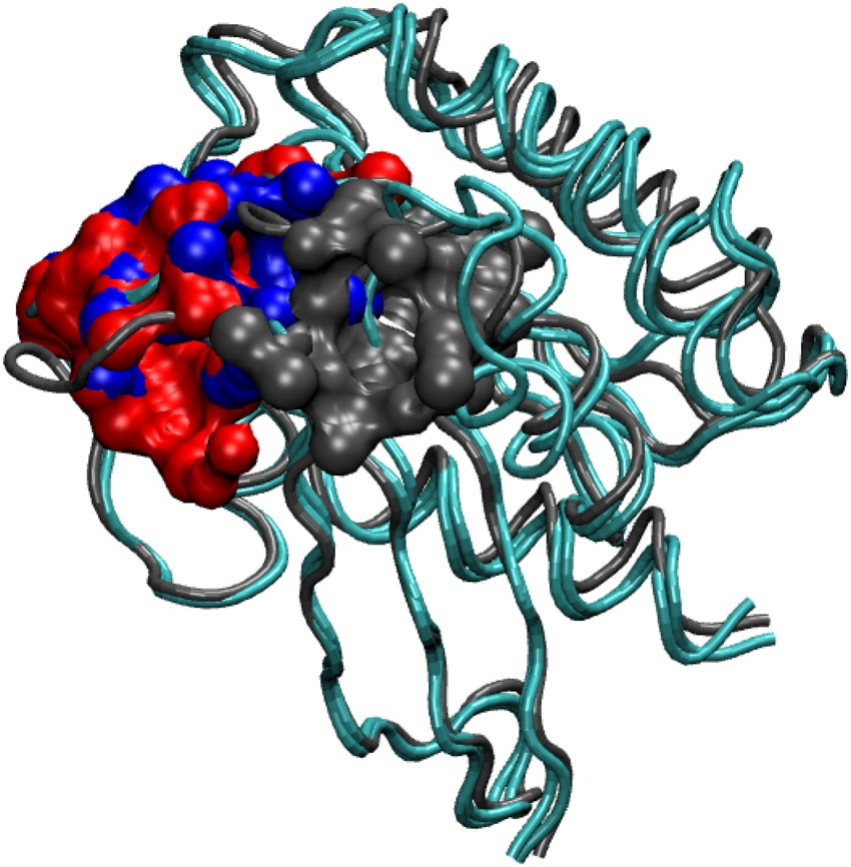
Largest cavity location of Rap domain in original crystal structure and last 5 ns averaged structures of GTP (red; volume: 605.96 Å^3^), Crystal structure(blue; volume: 521.01 Å^3^) and GTP-PSER11(black; 278.01 Å^3^) cases.

The conformational changes in Rap, observed both in terms of interactions between various loop regions via covariance analysis and overall conformational flexibility of the protein, prompted us to look for phosphorylation induced changes in the distribution of pockets within the protein. The results are shown in Fig. 9. The largest pocket in the Rap protein, by volume, is identified (averaged over last 5 ns of simulation) and the results are shown for Rap-GTP with and without phosphorylation and the original crystal structure are also shown for comparison. The pocket location remains more or less the same for all the forms of Rap protein, except when Rap is phosphorylated at SER11 and with ligand GTP (shown in dark grey in fig Fig. 9). As can be seen from Table S2, the residues lining the pocket are very similar in all the three forms of Rap-PSER11. Most of the residues that line the nucleotide pocket, not surprisingly, belong to Switch I, Switch II and P-loop (as seen from the colouring of the residues in Table S2). However in the case of GTP-PSER11, the residues are predominantly only from P-loop and Switch II and the Switch I loop residues are not part of the residues lining the nucleotide pocket, which can be rationalised in terms of increased mobility of Switch I loop. The pocket volume is also changed and is much smaller for the phosphorylated case. As discussed earlier, the change in the pocket location can be rationalised in terms significant perturbation to the nucleotide binding pocket of the Rap protein upon phosphorylation. The results clearly show that the location of the largest pocket is near the nucleotide in all the cases except when the protein is phosphorylated in the presence of GTP (GTP-PSER11 case). The observation that the largest pocket size in the case of phosphorylated Rap protein, in the presence of GTP, is smaller than all the other case, suggests a more compact structure when phosphorylated and in the presence of GTP which is consistent with the PCA results in Fig. 8.

### Effects of phosphorylation on complex and interactions at the interface

In this section we describe in depth the effect of phosphorylation on the interaction between Rap and Raf. Studies have shown that one of the functions of Rap proteins is to bind to the Raf effector via the RBD domain effectively trapping the Raf protein in an inactive complex^24^. The interaction between Ras and Raf is essential for activation of Raf kinase domain (which is located in the C-terminal half of the Raf protein), which plays a crucial signalling role in the MAPK pathway and Rap proteins can interefere with this mechanism by making Raf unavailable to Ras proteins. Proteins in Ras superfamily, including Rap protein, interact with effector proteins like Raf via Switch I loop^10,56^. Experimental studies^63^ have shown that single mutation of a conserved residue like THR 35 can significantly alter the dynamics of the Switch I loop and consequently affect the interaction of Ras superfamily proteins with the effector proteins like Raf. Switch I region (also called effector loop) is identical between Ras and Rap proteins. As seen in previous section, phosphorylation of SER11 residue leads to considerable changes in the mobility of the two main functional loops: Switch I and II of Rap protein which liganded with GTP. RMSF of Rap protein in the presence of GTP ligand shows that, the mobility of the Switch II and Switch I loop decreases and increases respectively (Fig. 2). Protein-protein binding exploits the inherent flexibility of the proteins to undergo conformational changes and form a complex^64,65^. In this section we explore the changes to the interface of Rap with Raf when SER11 of Rap is phosphorylated.

**Figure 10:**
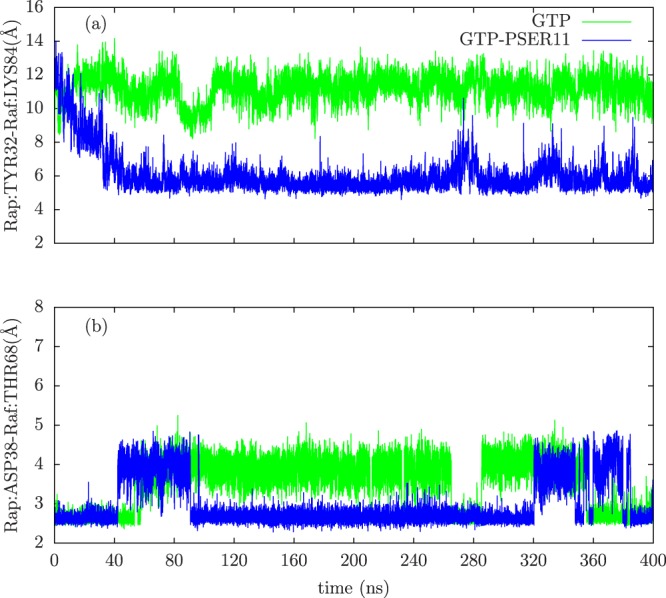
The time evolution of (**a**) distance between Rap:TYR32(CA) and Raf:LYS84(CA) atoms, (**b**) distance between Rap:ASP38(OD1) and Raf:THR68(OG1) atoms.

**Figure 11:**
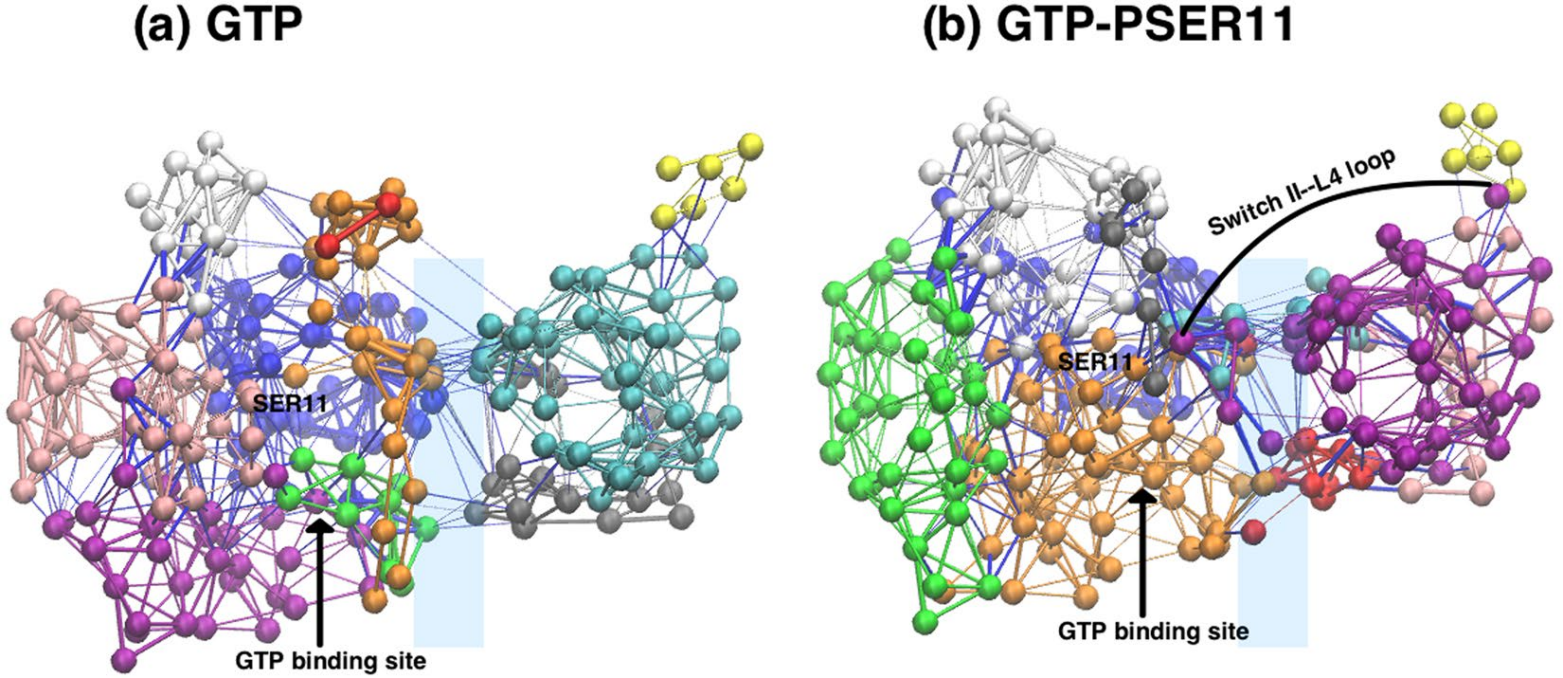
The communities detected in (**a**) GTP- and (**b**) GTP-PSER11 cases. The yellow community represents the L4 loop.

Unlike the majority of protein-protein interfaces which have more hydrophobic contacts^66^, the crystal structure of Rap-Raf suggests that there are many polar interactions between the Switch I loop of Rap and RBD of Raf^57^. The Switch I loop is not in close contact with the nucleotide binding pocket region in Rap protein but residue TYR32 and its conformational changes when GDP is exchanged for GTP, plays a crucial role in binding of the Switch I loop with the RBD of Raf protein^16^. This conformational change in Tyr 32 presumably facilitates the formation of a polar contact between residues ASP38 of Rap and ARG89 of Raf. As shown in previous section, the phosphorylation of SER11 significantly perturbs and reorganizes the nucleotide binding region and the surrounding loop conformations (See Figs 3 and 5). Due to breaking of strong polar interaction between GLU 30 and hydroxyl groups attached to ribose moieties of GTP ligand on phosphorylation of SER11, the Switch I acquires additional mobility and the loop moves away significantly compared to the unphosphorylated Rap-GTP case (See Fig. 3). This movement of Switch I loop, on phosphorylation, results in introduction of additional interactions between RBD of Raf protein and Switch I loop. Figure 10(a) shows the evolution of distance between TYR32 of Rap and LYS84 of Raf and in the case of phosphorylated Rap-GTP protein, the movement of the Switch I loop decreases the distance between Switch I loop and LYS84 of Rap by more than 6 Å. The consequences of such movement can be seen in the polar interactions between the RBD of Raf and residues of Rap at their interface (Fig. 10(b) and in Fig. S4). The time evolution of distance profiles suggest that few polar interactions like SER39-ARG89, GLU37-ARG59, ASP33-ARG84 remain unchanged with phosphorylation of SER11. However phosphorylation leads to changes in other polar interactions at the interface with disruptions in ASP33-ARG73 and GLU37-ARG67 interactions and enhancement in GLU54-ARG67 interaction. The polar interactions involving ASP38 of Rap are required interactions for effector binding to the Rap protein^16^ show positive enhancement upon phosphorylation. As can be seen in Figs 10(b) and S4(c), in phosphorylated Rap case, the ASP38 residue forms long-surviving ionic interactions with both ARG89 and THR68 residues of RBD of Raf protein suggesting increased binding between Rap and Raf with phosphorylation. Allosteric network analysis confirms this enhanced interaction (Fig. 11).

From the allosteric network analysis, we see that the number of detected communities remain the same (i.e 10 communities). However, the organizationof the communities at the interface is drastically different when Rap is phosphorylated. The most striking aspect of the network analysis is that the GTP-PSER11 case has atleast 3 communities in common to the interface between Rap and Raf proteins (cyan, purple and red communities in Fig. 11(b)), which is absent in the unphosphorylated case. New community (shown in purple in Fig. 11(b)) that connects the Switch II loop with the L4 loop of the Raf protein (which is closer to the Cysteine Rich Domain part of Raf), via the protein-protein interface emerges in the phosphorylated form. In a recent combined experimental and simulation study on a Ras-Raf complex, a similar result was obtained due to mutation of GLN61LEU^58^. The mutation resulted in altering the allosteric pathways in which a single community network was found to form between the interface of Ras-Raf complex and the distant L4 loop of Raf. The present observations of global effects of phosphorylation of a single residue SER11 in Rap protein reiterates the fact that such changes can be allosterically communicated to spatially distant regions in the complex and suggest how a local, single residue phosphorylation can have global effects.

### Effects of phosphorylation on binding energy of the complex

In the previous sections, we described how the phosphorylation of SER11 affects the dynamics of functional Switch I and Switch II loops and consequently how interacting communities spanning the complex interface emerges. In this section we look at the effect of phosphorylation on the binding free energy of the Rap-Raf complex, in particular to gain insight into the emergence of community across the complex interface. To do this, we used the standard MM-GBSA technique, as described in the Methods section. The results of the MM-GBSA calculations are shown in Table 1.

**Table 1:** Free energy contribution of GTP liganded simulations.

Contribution	GTP-SER11 system (Kcal/mol)	GTP-PSER11 system (Kcal/mol)
Δ*G*_*bind*_	−62.88 ± 8.59	−68.67 ± 8.35
Δ*E*_*elec*_	−498.66 ± 109.20	−520.18 ± 61.72
Δ*E*_*vdW*_	−45.88 ± 5.69	−48.48 ± 5.92
Δ*G*_*solv*_	481.66 ± 104.23	500.00 ± 57.16
Δ*G*_*solv−np*_	−8.95 ± 0.54	−10.01 ± 0.51
Δ*G*_*solv−polar*_	490.62 ± 104.17	510.02 ± 57.19

The binding energy values show that Δ*G*_*bind*_ of the complex is lower when the SER11 is phosphorylated suggesting an increased binding of Rap-Raf complex. It can be seen that the contribution to the increased binding energy of the complex has main contribution from the and Δ*E*_*elec*_ and Δ*G*_*solv*−*polar*_ terms. The binding free energy results and its contributions are consistent with increased interactions at the interface as seen in formation of long-surviving ionic interactions between ASP38 residue of Rap with both ARG89 and THR68 residues of RBD of Raf protein in Fig. 10 and also the emergent community at the complex interface shown in Fig. 11(b). The predominant contribution of electrostatic interactions to the binding free energy, seen here, is consistent with earlier work on thermodynamic analysis of Ras/Effector Complex Interfaces^67^. The entropy calculations were performed to understand any possible effects of phosphorylation of SER11, using the quasi-harmonic approach, and are shown in Table S2 of Supplementary Information. The calculations clearly suggest that effects of phosphorylation on the overall complex is minimal but, the Rap protein is less dynamic when SER11 is phosphorylated. This result is consistent with our PCA results, which suggests that the conformational sampling of the Rap protein is smaller when SER11 is phosphorylated.

## Discussion

The focus of the present paper is to understand the effects of phosphorylation of a single residue SER11 in Rap on the interactions between Rap and RBD of Raf and speculate on how network of new interactions, that emerge as a consequence of phosphorylation, are used to communicate the changes at the interface to the distal region of Raf. The SER11 phosphosite is present in the P-loop (see Fig. 1), close to the Switch I region and is relatively buried. This phosphorylation was identified earlier in an independent experimental study^49^. The mobility of the Rap protein, and in particular those of various functional loops, as seen from the simulations here suggest that the dynamic nature of the loop regions can potentially expose this buried phosphosite. Indeed solvent accessible surface area (SASA) measurements of SER11 residue in a 400 ns long simulation (when unphosphorylated) and with GTP ligand revealed that for a significant time on the simulation time scale, the SASA value of SER11 is greater than that of its crystal structure value [See Fig. S3]. This also corroborates the observation in Fig. 2 that the P-loop region is marginally dynamic. These two observations together lend credence to our assumption that though the phosphosite is buried in the crystal structure, the inherent protein dynamics can potentially expose the site to solvent and other relevant kinases which can phosphorylate the SER11 residue. Further simulations of the SER11 Phosphorylated GTP bound Raf Rap complex helped us to understand the effects of the phosphorylation on the possible global conformational changes in Rap protein, its interface with RBD of Raf and possible allosteric effects transmitted to spatially far away locations.

All of the observed changes upon phosphorylation converge on one unifying theme: SER11 phosphorylation stabilizes the GTP bound Rap structure, likely to prevent GTP hydrolysis by pulling and closing the Switch II loop over the active site and establishes an allosteric network that can potentially transmit these changes to the distal L4 loop of Raf. Furthermore the PCA results indicated that the conformational space occupied by this SER11 GTP bound Rap is dramatically different from the unphosphorylated form and reflects a tighter conformation of the protein. The simulations also reveal that there is significant alteration in the pocket location and its size when the SER11 residue in Rap is phosphorylated. This new pocket is flanked predominantly by residues in Switch II loop (and from P-loop), and is different from the unphosphorylated form in which the largest pocket is surrounded by Switch I loop residues. The allosteric network analysis suggests that there is an increase in communities across the interface with phosphorylation. More significantly a single community spanning residues in the Switch II loop all the way to distal L4 loop emerges on phosphorylation. The net effect of SER11 phosphorylation is an allosteric relay of signals from Switch II region in Rap to L4 loop in RBD of Raf kinase which could result in constitutive activation of Rap and consequently that of Raf kinase potentially affecting the downstream signalling. A recent work on Hsp90 family of proteins also suggested that such buried post translational modification sites can play an important role in allosteric conformational changes and can potentially act as mediators of global dynamics in the Hsp90 structures^68^. The binding free energy calculations concur with the other results and show that the binding of Rap, with GTP ligand, with RBD of Raf is stronger when SER11 is phosphorylated, though the difference may not be very strong given the possible errors in such calculations. This plausible increased binding has its origin in favourable electrostatic interactions between residues of the two proteins at the interface due to cascading effects of phosphorylation of SER11 and can have important consequences in the downstream MAPK signaling pathways. One of the functions of Rap proteins is to competitively bind to Raf, without activating it, and disrupt the Ras-Raf binding^20^. Increased binding of Rap to RBD, due to phosphorylation of SER11 residue, can potentially make Raf even less available to Ras. In addition, such phosphorylation is also seen to induce possible long-range allosteric communication between Rap (via Switch II) and L4 loop of RBD of Raf which connects RBD to the cystein rich domain (CRD) of Raf. The entropy calculations suggest that the phosphorylation of SER11 does not change the overall entropy of the complex significantly but when considered individually, the Rap protein has lower entropy inits phosphorylated state. This result is quite consistent with our PCA results and together they suggest that the Rap protein has a ‘tighter’ conformational sampling when phosphorylated. This possibly due to increase in favourable electrostatic attractions within the Rap protein, which are are cascadng effects of phosphorylation of SER11. Whether such increased binding of phosphorylated Rap wth RBD of Raf can trigger conformational changes in the CRD of Raf, further affecting the binding of Raf to Ras, since it has been suggested that CRD of Raf also binds to Ras^23,69–72^, is open to speculation.

There is a significant parallel between these changes observed in the present MD simulations with those of Ras protein GTP bound crystal structure when GLN61 is mutated to a LEU. This mutation is a well known oncogenic mutation that prevents GTP hydrolysis locking Ras in a constitutively active form^58^. The authors predicted that the extended long range allosteric effect transmitted across the interface to the L4 loop is responsible for the kinase activity of Raf. The similarity between the two observations strongly suggests that the phosphorylation at SER11 mimics the oncogenic mutation in Ras which when extrapolated to function suggests that Rap may be constitutively activated by such phoshorylation. This SER11 phosphorylation in Rap was observed in Hela cells when the EGFR is activated. This phosphorylation increases upon nacodazole treatment^49^. Other highthroughput studies have detected the same phosphoryation in tumors^51^. Although this phosphorylation does not seem to occur at high enough occupancy, it is nevertheless detected with high confidence. These observations suggests a likely scenario. It is possible that this phosphorylation of Rap happens in normal cells during ligand binding to receptors. It is also possible that aberrant signalling due to some over active kinase often seen in cancers may phosphorylate Rap and activate it. Consequently this may lead to disruption of Rap-Mapk signaling or it may independently activate other RBD domain containing effector proteins. Such a possibility is supported by the significant parallel between the observations reported here and those of Ras protein GTP.

In summary we believe that this case study is an example as to how integrating tools that can probe dynamics can yield wealth of biological information hidden in crystal structures and highthroughput studies. They can provide probable mechanism by which single site PTM or point mutations affect functions of a protein. In addition these results reveal new binding pockets in proteins not evident in static crystal structures but evolve due to dynamic changes in proteins. Such dynamic pockets may be trapped by small molecules to inhibit the functions of the protein thus expanding the repertoire of druggable genome space. Arguably one may target the kinase responsible for the phosphorylation of such proteins thus providing alternative strategies to inhibit the functions of notoriously un druggale and elusive protein such as the Ras GTPases.

## Methods

### MD Simulations

The effects of phosphorylation on the dynamics of complex Rap-Raf are studied using the available complex structure of Ras-related protein Rap1A (referred to as Rap henceforth) liganded with a GTP-analogue molecule, Mg^2+^ ion and RBD (Ras binding Domain) region of Raf protein variant c-Raf1 (referred to as Raf henceforth) downloaded from protein data bank with PDB id 1C1Y with a resolution of 2.2 Å^57^. All-atom classical MD simulations were done for the systems listed in Table S1, using CHARMM36^73,74^ forcefield with the aid of NAMD^75^ software. The visualization was done using software VMD^76^ and analysis of data using Tcl scripting language which is embedded with VMD, Matlab and Grcarma^77^ software. The protonation states for all ionizable residues were determined using PDB2PQR server^78^ before solvating them. Each system was solvated in a water box (using TIP3P^79^ water model) and overall charge neutrality was achieved through the addition of appropriate counter ions. The systems were then subjected to energy minimization runs using the conjugate gradient method for 5000 steps, followed by MD simulation runs in NPT ensemble. The GTP analogue in the crystal structure,GppNHp was replaced by GTP and GDP molecules for the present simulations. The simulations of Rap-Raf complex with GTP and GDP ligands with and without SER 11 phosphorylated were done for 400 ns with a time step of 2 fs. The Nosé-Hoover-Langevin piston with a decay period of 100 fs and a damping time of 50 fs was used to maintain a constant pressure of 1 atm^80,81^. Berendsen thermostat^82^ was used to control temperature at 298 K. A cut-off distance of 12 Å was used to compute all short-range van der Waals (VDW) interactions and the long-range electrostatics interactions was treated with the Particle Mesh Ewald(PME) method^83,84^.

### Covariance Analysis

Covariance Analysis is done on Rap-Raf complex C_*α*_ atoms of liganded cases to analyze the coupling between residues. The covariance matrix constructed from the displacements with respect to the average structure can give information regarding correlated/anti-correlated movements between various regions of the complex. The covariance matrix is constructed for the last 50 ns of the 400 ns long simulations. The covariance matrix is defined as following:

1 $${C}_{ij}=\frac{\langle {\rm{\Delta }}{r}_{i}\cdot {\rm{\Delta }}{r}_{j}\rangle }{\sqrt{\langle {\rm{\Delta }}{r}_{i}\cdot {\rm{\Delta }}{r}_{i}\rangle \langle {\rm{\Delta }}{r}_{j}\cdot {\rm{\Delta }}{r}_{j}\rangle }}$$

where 〈⋅〉 stands for the averaged values, Δ*r*_*i*_ and Δ*r*_*j*_ are the i^th^ and j^th^ atom’s displacements with respect to the corresponding averaged structure atoms.

### Principal Component Analysis

Molecular dynamics data is inherently high dimensional in nature and Principal component analysis (PCA) is therefore used to reduce the data into linear dimension for analyzing MD trajectory data^85–88^. The aim of this technique is to transform the coordinate system of the data such that the most variance is captured in least number of coordinates(principal axes). This representation is found by computing the eigen value decomposition on the covariance matrix of the data. The resulting coordinate vectors are the principal components. The largest eigen value corresponds to the most significant eigen vector (or first principal component) and the second largest eigen value corresponds to second principal component(PC) axis and so on. The nth eigen value represents the variance corresponding to the respective principal axis and the information content captured by nth PC is measured by the following expression:

2 $${I}_{n}=\frac{{\lambda }_{n}}{\sum _{n=1}^{3N}\,{\lambda }_{n}}$$

We apply PCA to M × 3N dimensional MD trajectory where M is the number of frames (which are the time snapshots from MD trajectory) and N is the number of C_*α*_ atoms (3N because of coordinates). In most of the cases we can capture about 70% of the information in the first very few (like 3 to 10) principal components. PCA was done on the cartesian coordinate data of C_*α*_ atoms from MD trajectory files (for Rap and Raf chains under different conditions) produced from simulations. This approach enables us to deconvolute the information available in MD trajectories and visualize how the proteins sample the conformational space.

### Pocket Identification

We used CASTp server^89^ for pocket identification in the Rap domain. The largest cavities of the last 5 ns time averaged structure of Rap domain for 4 different cases (as in Table S2) were computed. These calculations can give information about the flexibilty of the protein cavities and provides the necessary insights into comparative analysis of different forms of the protein as well as how each simulation is different from the original starting structures.

### Community Network Analysis

Community network analysis is a unique tool to identify possible signaling pathways between distant yet functionally relevant protein sites based on topology of communities that are derived from the long molecular dynamics trajectories^90–94^. The community network analysis on the MD trajectory data is done using *NetworkView*^90,95^ plugin in VMD. A network is a set of nodes connected using edges. Each C_*α*_ atom of amino acid in the protein is represented as a node here. Edges connect pairs of nodes if the corresponding residues are in contact, and 2 nonconsecutive monomers are said to be in contact if any C_*α*_ atoms from the 2 monomers are within 4.5 Å of each other for at least 75% of the frames (corresponding to the last 50 ns MD simulation trajectory of GTP- and GTP-PSER11 case) analyzed. The edges are weighted using correlation matrix(C_*ij*_) data between the C_*α*_ atoms using the relation:

3 $${w}_{ij}=-\,log(abs({C}_{ij}))$$

These weights in the form of correlation matrices are calculated using Carma software^96^. The correlations in the residue motion is used as a measure for information transfer between the two residues in contact. The suboptimal path is defined as the length of a path *D*_*ij*_ between 2 distant nodes *i* and *j* is the sum of edge weights between the consecutive nodes (*k*,*l*) along the path:

4 $${D}_{ij}=\sum _{kl}\,{w}_{kl}$$

The shortest distance between all pair of nodes is found using Floyd-Warshall algorithm^97^. The average of all shortest paths, known as critical path length (CPL), is a measure of the network size. The community detection analysis was done using software “gncommunities”^90^.

### MM-GBSA for Binding free energy measurements

MM-GBSA(Molecular Mechanics-Generalised Born Surface Area)^98–101^ calculations were done to estimate the binding free energy of Rap-Raf complex. MM-GBSA method has been used in many studies to calculate the binding free energy of ligands with biomolecules as well as between biomolecules^102–104^. This method is computationally cheaper than other free energy methods such as steered molecular dynamics^105^, free energy perturbation and metadynamics. In MM-GBSA method, the free energy of binding of either two proteins or a ligand a protein is obtained by calculating the sum of difference between the gas-phase molecular mechanical, the solvation free energy and the entropy terms. In the present work, we follow the single-trajectory method of simulating the entire complex and then decomposing the required trajectories out of it, which has been used in earlier works and has also been shown to yield more accurate results of free energy of binding than three separate simulations involving the complex and the two proteins^99,106,107^. In this work, the MM-GBSA analysis was performed on three subsets: the Rap-Raf complex, only Rap and only Raf for both GTP-SER11 and GTP-PSER11 systems and all the GBSA calculations were performed on these three trajectories for the last 100 ns (300–400 ns or 5000 frames) of simulations using NAMD^75^ software. In all the cases, water and counterions were removed for the calculations and equivalent implicit solvent model parameters were used in the NAMD. The free energy was calculated using the equation below:

5 $${G}_{TOT}={H}_{MM}+{G}_{solv}-T{\rm{\Delta }}{S}_{conf}$$

where *H*_*MM*_ is the sum of the bonded, electrostatic and Lennard-Jones energy terms, *G*_*solv*_ is the sum of polar and non-polar solvation energies,*T* is the temperature and *S*_*conf*_ is the configurational entropy. *G*_*solv*_ is included in the electrostatic measurements done via NAMD script for GBSA calculations. In the single trajectory method, employed here, the bonded energy contribution to the change in *H*_*MM*_ will be zero. Regarding the inclusion of entropic term in the free energy of binding, we would like to note that the usual method of computing the entropy via normal modes^108^ is computationally very expensive, especially for protein-protein complexes and other methods may have convergence issues and has been omitted in many earlier works, which we follow in this work as well^98,99,101,109–118^. The binding free energy for complex formation is then calculated as:

6 $${\rm{\Delta }}{G}_{bind}={G}_{TOT}^{Rap-Raf}-({G}_{TOT}^{Rap}+{G}_{TOT}^{Raf})$$

where ${G}_{TOT}^{Rap-Raf}$, ${G}_{TOT}^{Rap}$ and ${G}_{TOT}^{Raf}$ are the free energies corresponding to Rap-Raf complex, only Rap and only Raf trajectories. In addition to the binding free energy measurements, we also analysed the change in entropy of the individual as well as the complex using quasi harmonic approach implemented in the Wordom program^23,32,49,51,99,101,119,120^.

## Electronic supplementary material

Supplementary Information
