# Supplementary material for: Phosphorylation promotes binding affinity of Rap-Raf complex by allosteric modulation of switch loop dynamics
Source: Sci Rep. 2018 Aug 28;8:12976. doi: 10.1038/s41598-018-31234-7 (PMC6113251; doi:10.1038/s41598-018-31234-7)
Supplement: Supplementary file 1 — Supplementary Information [file 41598_2018_31234_MOESM1_ESM.pdf]

SUPPORTING INFORMATION:

Phosphorylation promotes binding affinity of Rap-Raf complex by allosteric modulation of switch loop dynamics

Devanand T[1,3], **Prasanna Venkatraman** [2,3,+], **and Satyavani Vemparala**[1,3,*]

[1]The Institute of Mathematical Sciences, C.I.T. Campus, Taramani, Chennai 600113, India
[2]KS 137, ACTREC Sector 22, Kharghar Navi Mumbai - 410210, India
[3]Homi Bhabha National Institute, Training School Complex, Anushakti Nagar, Mumbai 400094, India
[+]vprasanna@actrec.gov.in
[*]vani@imsc.res.in

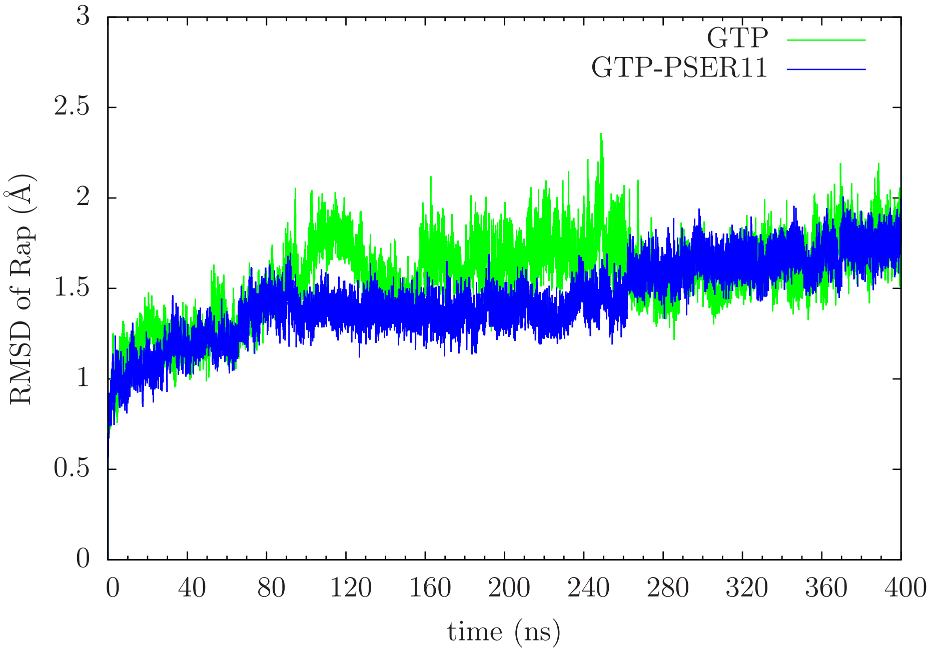

Figure S1. Time evolution of root mean squared deviation(RMSD) of Rap with GTP ligand with and without SER11 phosphorylated. Only C_α atoms of Rap chain are considered for computing RMSD.

No.	Description	Length of Simulation
1	Rap-Raf complex with GTP and Mg^{2+}	400ns
2	Rap-Raf complex with GTP and Mg^{2+} with phospho-Serine11	400ns
3	Rap alone with GTP	200ns
4	Rap with Phospho-serine11 with GTP	191ns

Table S1. Details of systems simulated and analysed within paper.

Selection	GTP-SER11 system		GTP-PSER11 system	
	ΔS (kcal/K/mol)	T ΔS (kcal/mol)	ΔS (kcal/K/mol)	T ΔS (kcal/mol)
Rap-Raf complex	1.7842	531.6916	1.7845	531.781
Rap chain	1.1707	348.8686	1.1624	346.3952
Raf chain	0.5978	178.1444	0.6137	182.8826

Table S2. Quasiharmonic entropy contribution of GTP liganded simulations.

No.	System-Name	Residues lining the largest pocket	Pocket Vol.
1	Rap-crystal structure: 1C1Y.pdb	GLY12 GLY13 VAL14 GLY15 LYS16 SER17 ALA18 PHE28 VAL29 GLU30 LYS31 TYR32 ASP33 PRO34 THR35 ASP57 THR58 ALA59 GLY60 THR61 ASN116 LYS117 ASP119 ALA148 LYS149	521.01 $\AA^3$
2	GTP Case	SER11 GLY13 VAL14 GLY15 LYS16 SER17 ALA18 VAL21 PHE28 VAL29 GLU30 LYS31 TYR32 ASP33 PRO34 THR35 ASP38 TYR40 ASN116 LYS117 ASP119 SER147 ALA148 LYS149	605.96 $\AA^3$
3	GTP-PSER11 Case	VAL8 LEU9 GLY10 LYS16 ILE36 GLU37 ASP57 THR58 ALA59 GLU62 GLN63 PHE64 MET67 ARG68 TYR71 LEU96	278.01 $\AA^3$

Table S3. The systems for which the pocket analysis is done. Other than for crystal structure, the pocket analysis was done on averaged strucutre of 395 to 400ns of trajectory data. The color code used is red for P-loop, blue for Switch I and green for Switch II residues.

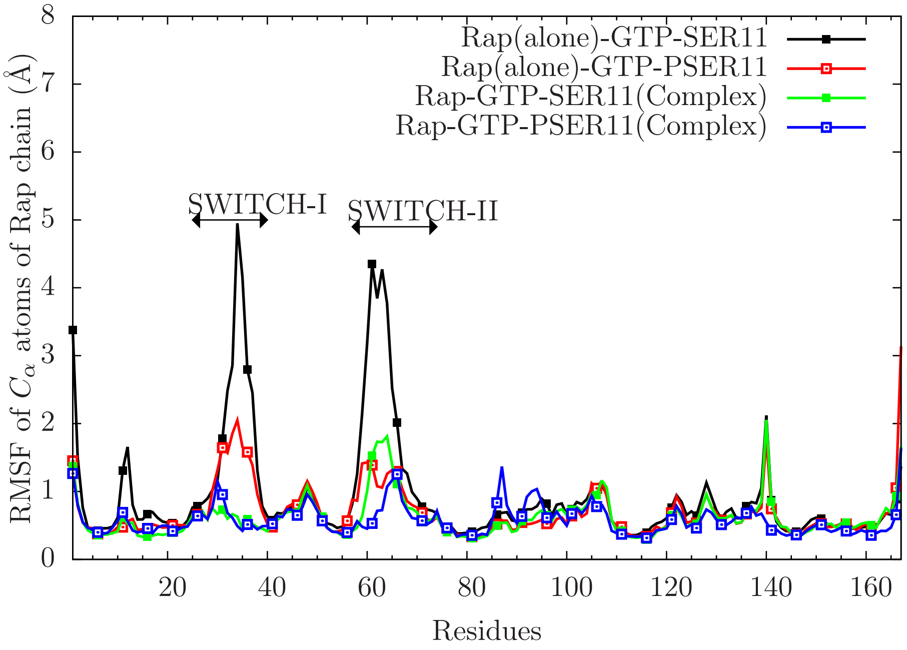

Figure S2. RMSF of Rap chain carbon-alphas corresponding to the last 50ns of MD trajectories.

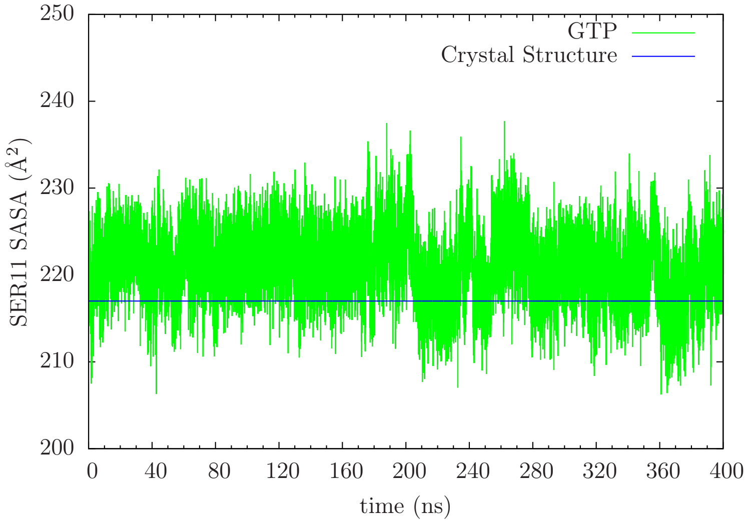

Figure S3. Time evolution of SASA value of unphosphorylated SER11 residue in Rap protein (with GTP ligand). For comparison, the SASA value of SER11 in the crystal structure is shown as blue line.

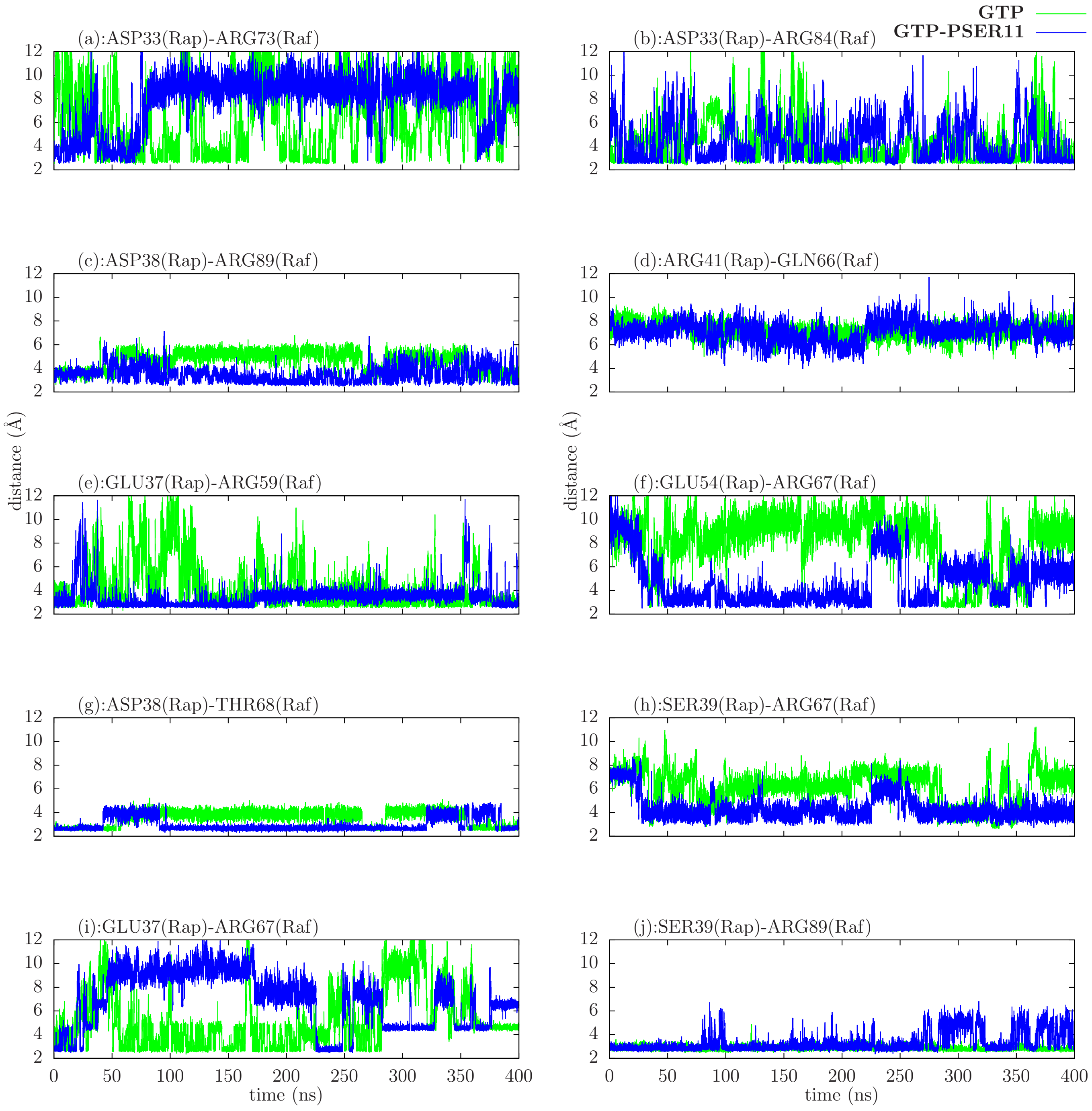

Figure S4. Time evolution of distances between critical residues at Rap:Raf complex interface (Rap bound with GTP) with and without SER11 phosphorylated.
